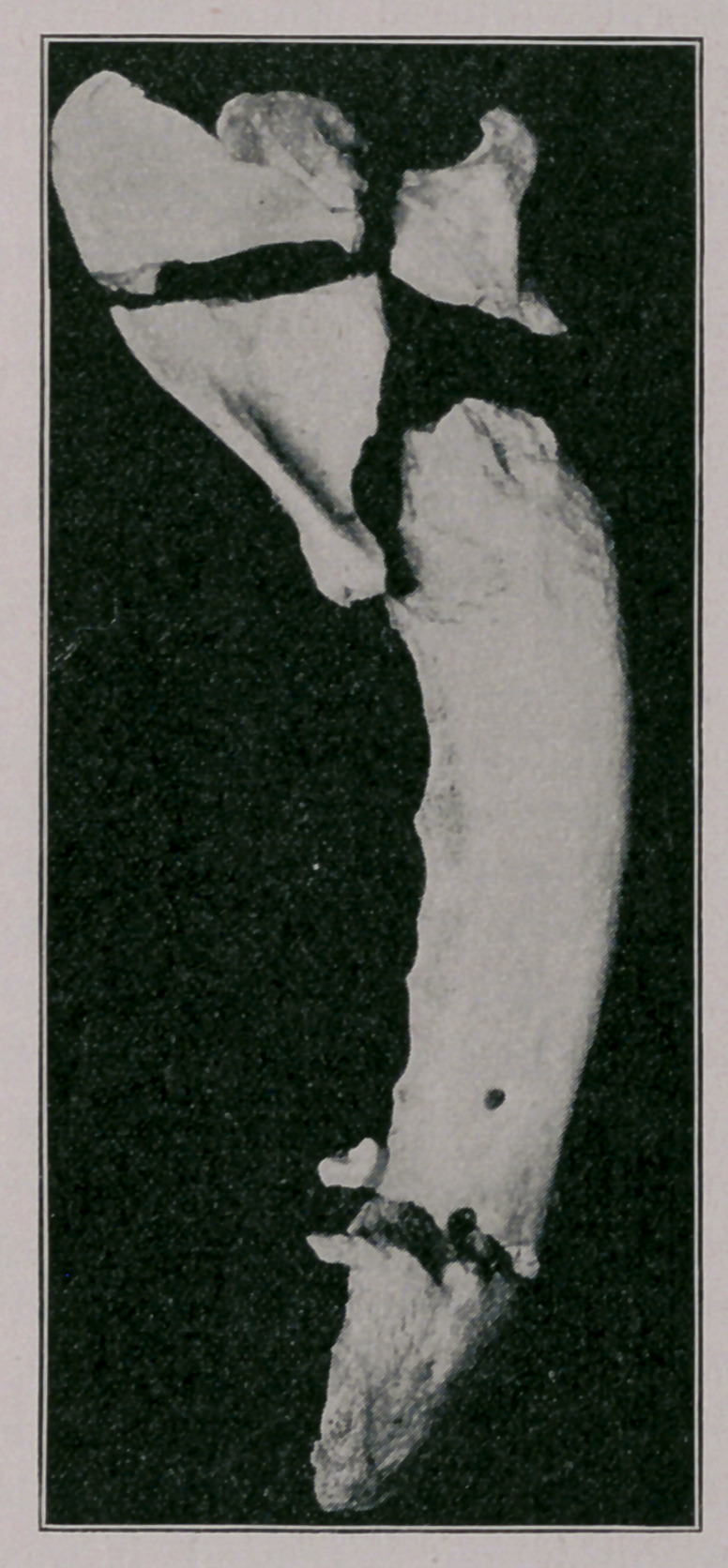# Department of Canine and Feline Medicine and Surgery

**Published:** 1901-01

**Authors:** Cecil French

**Affiliations:** Washington, D. C.


### DEPARTMENT OF CANINE AND FELINE MEDICINE AND SURGERY.

By Cecil French, D.V.S.,
WASHINGTON, D. C.

### Direct Compound Comminuted Fracture with Indirect Simple Fracture of the Inferior Maxilla.

The subject of this accident was a full-grown collie dog. The lesions were caused by a bullet from a rifle in the hands of a malicious person. An exact account of the happening could not be obtained, but it was known that but one cartridge was discharged, and consequently in estimating the manner in which the remarkable lesion was determined there was but one bullet to be taken into consideration. When the animal was seen by me, some twelve hours subsequent to the occurrence, it was in a dazed condition and walked with an unsteady gait. The head was held sideways, and the lower jaw was pendant and inclined laterally. The whole appearance was quite suggestive of paralytic rabies.

On closer examination I found two fractures existing in the left half of the inferior maxilla. One was situated just posterior to the root of the canine tooth. It was a transverse, simple fracture, having no visible communication with the air, although the surrounding textures were much discolored and tumefied. The second premolar was also involved, being split into two distinct portions.

The other fracture could be detected posterior to the last molar. At this point there was an orifice in the gum which permitted of the introduction of the little finger and through which splintered bone could be easily felt. In the cheek at about this level there was a more or less lacerated perforation.

Pulsations were about 120 and respirations slightly increased and somewhat labored. No other wound could be detected in any part of the body, though it must be remembered that, the animal being densely coated, a minute orifice might easily have been overlooked; hence my conclusion as to the course of the missile turned out to be faulty. I supposed that the animal had been struck a glancing blow by the bullet on the maxilla at the level of the second premolar while approaching the person with open mouth, and that the bullet had been then deflected to the angle of the jaw, causing a second fracture there, and had emerged at the spot of perforation in the cheek. That the bone did not happen to be exposed at the seat of the anterior fracture caused no wonder, as it is well known that deep organs often sustain a considerable degree of destruction from violence, while the superficial parts may exhibit hardly a trace of injury. I need refer only to run-over accidents, in which the integument and muscles of the abdomen may appear but slightly injured, while the liver, for instance, is extensively ruptured. That there was an orifice in the gum communicating with the splintered bone, and the only one apparently, called up in my mind a question as to the correctness of my conclusion; but it must be remembered that examination of this part was carried out under much difficulty, owing to the pain experienced by the animal at the slightest movement of the parts. Moreover, from time to time one hears accounts of such extraordinary courses taken by bullets that he is ever prepared to experience the unexpected.

Some four hours after the examination the dog died. Next morning I made a necropsy. On opening the abdominal cavity there was an escape of foul-smelling gas. The case was becoming more interesting. Proceeding, I found a considerable quantity of foul, bloody liquid free in the peritoneal cavity and evidence of recent acute peritonitis. I could not distinguish any free fecal matter. After I had exposed both thoracic and abdominal cavities it was easy to see what had happened. The bullet had passed down the neck and entered the thoracic cavity without leaving any external sign of its passage or damaging any important vessel. It had then perforated the left lung and diaphragm, lacerated one lobe of the liver, perforated the stomach, traversed the surface of the spleen, and fractured one rib. From this point I was unable to further trace its course, though it certainly did not emerge there. My conclusion that the wound in the cheek was the point of exit of the bullet was therefore wrong. Instead, it was the point of entrance, and the fact of the existence of the orifice in the gum at the level of the cheek wound became intelligible. The bullet coming in direct contact with the posterior portion of the bone had shattered it. How, then, did the anterior fracture occur? Certainly not by force exerted directly at that spot. It will be remembered that this region of the lower maxilla is most commonly the seat of fracture, it being the weakest portion of the bone. The symphysis also occasionally suffers, but this lesion is to be regarded more as a separation of the two component portions of the entire inferior maxilla than as a fracture. The symphysis is possessed of considerable elasticity, which tends to preserve it from the effect of violence exerted at a distant point. The only way to account for the anterior fracture in the case in question is to suppose that the force of the bullet exerted at the posterior extremity of the bone tended to bend it downward or inward to the extent that it snapped at its weakest spot, the symphysis acting as a fulcrum. I may add that I have questioned the authorities at the Army Medical Museum as to whether such a fracture is known in the surgical history of the Civil or Spanish Wars, but they have been unable to recall any incident characterized by similar phenomena.

Consequently, this fracture must be placed on record as unique and extraordinary.

## Figures and Tables

**Figure f1:**